# Gauteng mental healthcare providers’ understanding of cultural and religious illnesses

**DOI:** 10.4102/safp.v67i1.5779

**Published:** 2025-02-18

**Authors:** Ellen M. Mathapo-Thobakgale, Fhumulani M. Mulaudzi, Roinah N. Ngunyulu

**Affiliations:** 1 Department of Nursing Sciences, Faculty of Health Sciences, University of Pretoria, Pretoria, South Africa

**Keywords:** cultural and religious illnesses, understanding, phenomenology, hermeneutic, mental illness, mental healthcare providers

## Abstract

**Background:**

Cultural and religious illnesses such as spirit possessions are health conditions that are not easily understood by healthcare providers in mental healthcare service institutions. Mental health care providers’ understanding is guided by the Diagnostic and Statistical Manual of Mental Disorders, fifth edition (DSM-5) that seems to not recognise cultural and religious illnesses as a disorder that needs distinct care. The study explored mental healthcare providers’ understanding of cultural and religious illnesses that could assist spirit-possessed persons to receive proper management and early referrals to traditional health practitioners and faith healers who are expects in cultural and religious illnesses.

**Methods:**

Hermeneutic phenomenology explored 12 mental healthcare providers’ understanding of cultural and religious illnesses. In-depth individual interviews were conducted with 12 mental healthcare providers who were selected through a purposive sampling technique. Data were collected from two mental healthcare institutions in the Gauteng province of South Africa that provide mental healthcare services. Data analysis followed Heidegger’s and Gadamer’s philosophies and Van Manen’s six steps.

**Results:**

The findings revealed that mental healthcare providers understood cultural and religious illnesses as mental illness that is unclassified, a calling for a person to become a traditional health practitioner, a demonic spirit and/or witchcraft. The term ‘unclassified disorder’ denotes that there are no specific criteria that could be used to classify an illness.

**Conclusion:**

Understanding of cultural and religious illnesses could assist mental healthcare providers that ill-nesses that do not respond to psychiatric treatment can be referred to traditional health practitioners with expert cultural and religious assessment.

**Contribution:**

The study could assist MHCPs to acknowledge and take culture and religion into account when providing care to person with cultural and religious illness. Considering the culture and religion of the spir-it-possessed person could be an attempt to move towards a holistic understanding of health needs that highlight the continuous connections between mind, body, and soul.

## Introduction

Cultural and religious illnesses are a common and increasing health problem seen in countless communities globally which is still not clearly understood by healthcare providers, leading to a health concern in general. According to Lee,^1^ cultural or religious illness is a form of ritual practice and a cultural belief system in which the human being is viewed ‘as consisting of several elements such as body, mind, personhood, self, name, identity, soul or souls, even part souls’. In these cases, one or more of the person’s elements which could be body, mind, personhood, self, name, identity, soul or souls, even part souls may be replaced, temporarily or permanently, by another entity.^1^ Moscicke^2^ indicated that cultural and religious illnesses have increased globally in different regions of the world such as Africa, Asia and Latin America including modern cities like New York, Toronto, Paris and Cologne. Leistle^3^ understood cultural and religious illnesses as a disorder or an illness, that included pain, suffering and was characterised by the intrusion of the alien into the client’s experience. According to Boddy,^4^ cultural and religious illnesses are part of a daily experience that has to do with one’s relationship to the world and not just a dramatic ritual. However, Moscicke^2^ cited that cultural and religious illnesses are a struggle between God and Satan. According to Zaretsky and Leone,^5^ cultural and religious illnesses are a positive cultural tool that could be used to maintain order between humans and a supreme being. The distinct descriptions of the understanding on cultural and religious illnesses were based on the manifestations presented and observed by individual researchers. Sue, Sue, Sue and Sue^6^ reported that spirit possession manifestations were considered culturally normal by certain religious persons. At the same time, the mental healthcare providers (MHCPs) whose culture or religion differed from that of the client usually labelled it as ‘maladaptive’. Weiten and Hassim^7^ indicated the following manifestations that could be presented or observed from spirit-possessed persons: a history of behavioural changes, telepathic powers or levitation, vegetative symptoms, self-mutilation, hallucinations and/or feelings of being followed by their enemies. Sobekwa and Arunachallam^8^ reported that the manifestations could be because of the spirit possessing and capturing the mind and intellect of a client. Diagnosing such persons as mentally, physically or psychologically ill is difficult.

African and international MHCPs have clearly misunderstood the nature of cultural and religious illnesses. In support, Laher^9^ reported that in the Australian context, healthcare providers have limitations in understanding and treating indigenous Australians. Furthermore, Subu et al.^10^ revealed the need for MHCPs to gain an in-depth understanding of traditional, religious and cultural views about causes of mental illness and use of traditional and/or alternative treatments in the wider Asian context. This misunderstanding could be because of manifestations that mimic or are like manifestations found in other mental disorders.^11^ These similarities and misunderstandings could interrupt the provision and integration of cultural and religious support in long-term psychiatric care and mental health services.^5^

Sue, Sue, Sue and Sue^6^ reported 74% of the presence of cultural and religious illnesses cross-culturally since 1960s. In sub-Saharan Africa and the circum-Mediterranean, the estimation of belief is higher with 81% of cultural and religious illnesses being reported.^5^ Furthermore, an estimated 30% – 40% of cultural and religious illnesses globally were reported to be more in women than in men.^9^ The researchers identified a gap in the literature where it was noted that MHCPs did not always recognise that a person might have or is spirit-possessed instead of having a mental health issue. Having this understanding could assist MHCPs to recognise cultural and religious illnesses early.

The researchers observed that spirit-possessed persons who presented symptoms such as prophecies, visions, dreams, revelations, among others are on most occasions treated and managed as though the diagnosis is a mental disorder. In mental healthcare institutions, people suspected of cultural and religious illnesses are often misdiagnosed, mismanaged and poorly cared for. Misdiagnosis and mismanagement could be because of how MHCPs are trained and socialised in their profession. The statement was confirmed by other MHCPs who stated that some traditional health practitioners (THPs) have been prescribed physiological treatments based on the MHCP experiences in healthcare practice.^12^ The Diagnostic and Statistical Manual of Mental Disorders, fifth edition (DSM-5) criteria also lack a clear acknowledgement of the prevalence of some cultural and religious illnesses such as ancestral illness and/or calling, as is not yet recognised by psychiatric taxonomy as a scientific diagnosis.^13^

In support, van der Zeijst et al.^14^ revealed that the ancestral illness is regarded as a gift, not mental illness, in spite of the sufferings, warning signs and disturbances that accompany the calling at the onset. Furthermore, such cultural or religious illnesses were included in the fourth edition of the American Psychiatric Association’s Diagnostic and Statistical Manual of Mental Disorders (DSM-IV-TR) but were omitted from the subsequent fifth edition (DSM-5).^14^

Brenman, Luitel, Mall and Jordans^15^ as well as Wakefield^16^ reported that spirit-possessed persons did not meet their cultural and religious needs in the mental healthcare institutions as the DSM-5-TR provides clear, highly detailed definitions of mental health and brain-related conditions and disorders^17^ but not specifically spiritual illnesses such as calling or witchcraft. Instead, the spirit-possessed persons received treatment and care based on the mental healthcare provider’s clinical judgement and the DSM-5. Although the DSM-5 is used and considered to be the standard diagnostic instrument for diagnosing mental disorders, there is still a gap in assessing, classifying and explaining cultural and religious illnesses such as spiritual illnesses and possession among others.^18^ Misdiagnosing and mismanaging cultural and religious illness could progress to a chronic psychosocial state of illness or mental illness that could have been prevented by the MHCPs’ understanding of cultural and religious illnesses. Mental healthcare providers who understand cultural and religious illnesses are likely to assess and provide treatment and care accordingly. Furthermore, this study could assist MHCPs in acknowledging and taking culture and religion into account when providing care to spirit-possessed persons. Considering the culture and religion of the spirit-possessed person could be an attempt to move towards a holistic understanding of health needs that highlight the continuous connections between mind, body and soul.

### Study aims

This study aims to explore MHCPs’ understanding of cultural and religious illnesses to assist spirit-possessed persons to receive proper management and early referrals to traditional health practitioners and faith healers who are experts in cultural and religious illnesses.

## Research methods and design

The researchers used a phenomenological, hermeneutic research approach to explore MHCPs’ understanding of cultural and religious illnesses.

### Setting

The study took place at the two mental healthcare institutions located in Gauteng province, South Africa. These two mental healthcare institutions were chosen because they have enough advanced and basic psychiatric nurses and psychiatrists. One institution is situated in the western side of Pretoria whereas the other one is found on the western part of Johannesburg.

### Study population and sampling strategy

The study’s population consisted of 12 MHCPs (psychiatric nurses and psychiatrists) who have been working in mental healthcare institutions and providing care to mental healthcare users who have presented signs and symptoms of cultural and religious illnesses for 6 months or more. Purposive sampling was used to select the MHCPs. The study population were aged between 23 years and 65 years. Only MHCPs who were eager to talk about their understanding and agreed to provide rich and unique stories were included.^18^ The researchers included psychiatric nurses and psychiatrists who met the criteria and volunteered to participate as the sample size. The demographic details of the selected participants are presented in Table 1.

**TABLE 1:** Demographic data.

Characteristics	Mental healthcare providers
**Gender**	
Male	4
Female	8
**Age (years)**	
Male	29–60
Female	30–65 and above
**Race**	
African people	9†
White people	2‡
Indian people	1‡
**Religion**	
Charismatic	5
Apostolic	3
Christian	4
Months/years working with person with cultural and religious illness	6 months and above
**Total number of participants**	**12**

*Source:* Thobakgale EM. Spirit-possession as a mental illness: A phenomenological study in Gauteng Province [doctor of philosophy thesis]. Nursing Department, University of Pretoria; 2021

† , Psychiatric nurses;

‡ , Psychiatrist.

### Data collection methods

A pilot interview was conducted with two MHCPs (one psychiatric nurse and one psychiatrist) who were not part of the main study. Data collection was conducted in English as the medium of instruction. Following the pilot interview, the researchers refined the interview guide and added ‘Protestant religion’ before conducting the main interviews. The researchers, promoters and mental healthcare lecturers discussed the interview guide to ensure the strength, depth, richness and orientation towards cultural and religious illnesses.^19^ In-depth individual interviews were used to collect data. The researchers used a voice recorder to capture the data with the participants’ permission. A self-developed interview guide with one main question (i.e. *What is your understanding of cultural and religious illnesses*?) and probing questions were used. Data were collected from eight participants. Four additional interviews were conducted until saturation was reached at the 12th MHCP. Each interview lasted over an hour, 60 min to 86 min.

### Data analysis

The researchers used Van Manen’s^20,21^ six steps for thematic analysis adapted from the study conducted by Thobakgale.^22^ Furthermore, the researchers used Heidegger and Gadamer’s work to extend and deepen their thinking that focusses on the mental healthcare providers’ understanding of cultural and religious illness, namely:

- Step 1: Turning to the nature of the lived experience:
  ■ The researchers familiarised themselves with cultural and religious illnesses as the phenomenon of interest, formulated the research question and clarified assumptions and pre-understanding.
- Step 2: Investigating experience as we live it:
  ■ The researchers captured the phenomenon through methods of investigation such as interviews and use of personal experience as a starting point. Furthermore, they searched idiomatic phrases, obtained experiential descriptions from others, consulted phenomenological and experiential descriptions in literature and life stories as a resource from experiential material, diaries and journals.
- Step 3: Phenomenological writing/describing the phenomenon in the art of writing and rewriting:
  ■ The researchers made the feelings, thoughts and attitudes of the participants visible through the process of writing. The researchers focussed more on the MHCPs’ spoken language and thoughts of their understanding of cultural and religious illnesses. The researchers made a table of all the thematic statements and themes, then wrote notes and paragraphs on each to capture the themes. The researchers, study promoter and co-promoter repeatedly discussed repeated themes at regular intervals until the final themes were settled on.
- Step 4: Phenomenological reflection/reflecting on the essential themes, which characterise the phenomenon:
  ■ The overall meaning of the participants’ experience was sought when reflecting on the themes. The researchers conducted thematic analysis to seek meaning. Therefore, the reflection notes assisted during the data analysis.
- Step 5: Maintaining a strong and oriented position to the phenomenon:
  ■ To maintain a strong and orienting position on cultural and religious illnesses, the researchers used journal of reflection to refocus on the research question. They kept frequent contact with the colleagues and supervisors.
- Step 6: Balancing the research context by considering the parts and the whole:
  ■ The researchers constantly measured the overall design of the study and/or text against the significance that the parts must play in the total textual structure. Then, the data were put together sequentially to look at the whole picture to ensure that all the dissimilar parts contributed to the production of a complete picture. The assessment and reassessment processes were applied by reading and re-reading the data.^23^ Note that the interview transcripts were analysed immediately after the first data collection.

### Ethical considerations

The researchers obtained permission to conduct the study from the Department of Nursing Science’s in-house committee, the School of Health Care Sciences Post-Graduate Committee and the Department of Health in the Gauteng province and the National Health Research Database. The approval and clearance certificates (UPREC: 201/2018) were obtained from the Faculty of Health Sciences Research Ethics Committee. Further, the researchers obtained informed consent from the participants (i.e., the MHCPs) after giving thorough and truthful information about the study’s aim. The researchers also assured them that participation was voluntary and that they were free to withdraw any time after agreeing to participate in the study.^19^ The researchers ensured confidentiality by not recording the participants’ names and institutions where they were providing mental healthcare services. The researchers ensured the participants that the information they provided on the understanding of cultural and religious illnesses was confidential and would remain between them, the researchers and the promoters.

## Results

The study’s findings revealed that MHCPs’ training and education, culture and religion influenced their understanding of cultural and religious illnesses as a mental illness, ancestral calling, demonic power and/or unclassified disorder.

Understanding of cultural and religious illnesses as understood by MHCPs yielded the following themes:

- Theme 1.1: Cultural and religious illnesses as mental illness
- Theme 1.2: Cultural and religious illnesses as ancestral calling
- Theme 1.3: Cultural and religious illnesses as demonic power
- Theme 1.4: Cultural and religious illnesses as an unclassified disorder

### Theme 1.1: Cultural and religious illnesses as mental illness

Mental healthcare providers understood cultural and religious illnesses as a mental illness. The responses from the MHCPs were based on their own understanding and the training they received. Furthermore, the MHCPs understood cultural and religious illnesses in various ways including as psychotic disorders, perceptual disturbances, delusions and dissociative identity disorders (DID). Mental healthcare providers reported that cultural and religious illnesses had similar manifestations as mental illness, but that they were not the same.

#### Psychotic disorders

Mental healthcare provider 1 reported their understanding of cultural and religious illnesses as a psychotic disorder and said:

‘[*T*]here will also be other things to worry about such as threatening and/or yelling at others or [*physically*] by engaging in some type of violence. On the other hand, usually [*they*] turn out to be psychotic. They may have a disorganised behaviour and they will be out of keeping with usual behaviour.’ (MHCP 1, Psychiatrist, female)

#### Perceptual disturbances

Mental healthcare provider 1 stated:

‘[*T*]there is very vivid visual hallucination.’ (MHCP 1, Psychiatrist, female)

Mental healthcare provider 5 said:

‘Usually a person will display symptoms such as hallucination, say things like she sees [her deceased] grandfather speaking to her or seeing people coming to tell her [*things*].’ (MHCP 5, Psychiatric nurse, female)

#### Delusion

Mental healthcare provider 1 (psychiatrist) reported that the spirit-possessed persons believed that:

‘[*I*]in their mind they hear the voices of the ancestors telling them that they should follow the route of becoming a traditional healer.’ (MHCP 1, Psychiatrist, female)

Mental healthcare provider 7 said:

‘[*T*]hey will say they have the powers to heal people. There is also a similarity with the traditional aspects.’ (MHCP 7, Psychiatric nurse, male)

#### Dissociative identity disorders

‘[*T*]here is other alarm such as disorganised behaviour where you also have episode of amnesia.’ (MHCP 1, Psychiatrist, female)

‘I belief that there is no spirit possession, when the neurotransmitter in the brain are imbalanced, so if the dopamine level is very high may cause a person to have delusions, hallucinations or disorganized behaviour because of chemical imbalance.’ (MHCP 5, Psychiatric nurse, female)

‘For this topic, we do classify according to the mental health problems. Spirit possession as a health problem or a mental illness but it is amongst the traditions or beliefs that a patient might have. It is not classified as an illness as an entity but we say the mental illness might be caused by other factors which might include spirit possession, but not as a diagnoses, the spirit-possession.’ (MHCP 7, Psychiatric nurse, female)

### Theme 1.2: Cultural and religious illnesses as ancestral calling

Some MHCPs’ beliefs influence their understanding of cultural and religious illnesses as an ancestral calling that drove a person to train as a traditional health practitioner or faith healer following the process of *ukuthwasa*. Some of the MHCPs stated:

‘[*W*]hat we normally see is more of *ukuthwasa* [*training to become traditional health practitioner*], people who are called to become tradition healers.’ (MHCP 1, Psychiatrist, female)

‘[*C*]ultural and religious illnesses are also understood as a calling according to me, it may be religion … it is the normal way.’ (MHCP 3, Psychiatric nurse, female)

‘[*O*]ne is traditional practitioner, err may be he has gone through some rituals and training err to heal and may be, whatever training that the person [*healer*] gone through mostly is through spirits.’ (MHCP 4, psychiatric nurse, male)

‘That basically how I understand spirit possession, and sometimes you find that we say someone is spiritual with ancestral spirit and then maybe there is a great grandfather who was a traditional healer and that person needs to take up the practice of the grandfather.’ (MHCP 5, Psychiatric nurse, female)

‘[*I*]s a difficult or controversial subjects because errr, we know that there are people who are able to foresee or tell the future without them being actually disturbed that they do not need any kind of mental health services. And this will be people like your prophet in churches, sometimes *inyangas* and sometimes ordinary people on the street whom you will meet for the first time and they can actually tell you something you know it can be correct or true about yourself.’ (MHCP 8, Psychiatric nurse, male)

### Theme 1.3: Cultural and religious illnesses as demonic power

Mental healthcare providers understood cultural and religious illnesses as demonic power or as an attack of demons or Satan. The person who experienced demonic power presented strange behaviour, fought or performed hurtful acts uncontrollably under the influence or guidance of the possessing spirit as indicated in the quotes. Mental healthcare providers 3 and 6 said:

‘[*T*]he person might be having demonic-like possession and becomes aggressive.’ (MHCP 3, Psychiatric nurse, female)

‘Like both the evil and the Holy Spirit directs you but in different ways. The Holy Spirit direct you in a right way while the evil spirit direct you in an evil wrong way.’ (MHCP 6, Psychiatric nurse, female)

Mental healthcare provider 7 indicated that cultural and religious illnesses often manifested through a voice or body of a spirit and said that:

‘[*T*]he demon in the client would start talking strangely, saying I am a demon. It will say to the client don’t eat the whole day today. This shows that they have control over him.’ (MHCP 7, Psychiatric nurse, male)

Mental healthcare provider 10 further stated:

‘[*P*]ossession by a spirit is a bit of a difficult way to understand. Although yes we understand it that in the realm of the Satanic environment and in context of the word evil spirit. So I think you are quiet correct when you say spirit possession. I just use the term demon possession because this is the term I have been confronted with for the past 28 years in the psychiatric hospital.’ (MHCP 10, Psychiatric nurse, female)

### Theme 1.4: Cultural and religious illnesses as unclassified disorder

‘Unclassified disorder’ is understood to mean all types of mental illnesses that are not classified under DSM-5. Due to limited manifestations that meet DSM-5 criteria, cultural and religious illnesses are types of blurred conditions or disorders that are not clearly classified and written in the mental healthcare books or guides. These disorders do not meet the criteria to be diagnosed as mental illness or disorder. Mental healthcare provider 2 said:

‘[*C*]ultural and religious illnesses are a grey area, something that is very difficult and is not written in black on white.’ (MHCP 2, Psychiatrist, female)

Mental healthcare provider 9 reported that cultural and religious illnesses could not be classified as either physical, psychological, or emotional as they did not meet the classification criteria listed in DSM-5 and other guides such as the Cultural Formulation Interview:

‘I am actually not entirely sure, when that can be classified as a mental illness or whether that it is religious or something real, … I can’t be sure that it is a mental illness. Moreover, I do not think that it is mental illness, so I think there are people who may say they are possessed by spirit but are not mentally ill. They are not mentally ill, because there is a criterion which someone must meet to be classified as mentally ill and you find that most of the time, they do not really satisfy that [*criterion*].’ (MHCP 9, Psychiatrist, male)

## Discussion of findings

### Understanding cultural and religious illnesses as mental illness

Understanding how MHCPs in the mental healthcare institutions view cultural and religious illnesses can assist spirit-possessed persons in receiving holistic care and management. Cultural and/or religious background could influence MHCPs’ understanding of cultural and religious illnesses. In support of this, Delmonte, Lucchetti, Moreira-Almeida and Farias^24^ as well as Al-Adawi et al.^25^ reiterated that several authors consequently started to describe cultural and religious illnesses according to the prevailing social scientific paradigm. In the DSM-5, cultural and religious illnesses are classified as DID manifestation.^18^ In contrast, Vagrecha^10^ described cultural and religious illnesses as a culturally sanctioned, heavily institutionalised and symbolically invested means of expression in action of various ego-dystonic impulses and thoughts. Mental healthcare providers described cultural and religious illnesses as a disorder with psychotic features, perceptual disturbances, delusion or DID.

Mental healthcare providers reported cultural and religious illnesses as psychotic disorders wherein a person presented with behaviour that looked odd and disgraceful, had trouble in handling stressful situations, while others experienced visual and auditory hallucinations. Nyathi^13^ agreed that cultural and religious illnesses are sometimes understood as psychotic or mood disorders. In contrast, Rashed^26^ reported that cultural and religious illnesses were just invaders in a person’s life that caused physical and psychological illnesses but not mental illnesses. Furthermore, MHCPs understood cultural and religious illnesses as perceptual disturbance as evidenced by a person who sees, feels, tastes, hears and smells or senses things that could have happened. Sometimes the spirit-possessed person could sense that someone would be involved in an accident and the same thing happened according to the prophecy. Tracy and Shergill^27^ confirmed that perceptual disturbances could be auditory perceptions or verbal perceptions that are subjective perceptions of external speech in the absence of external stimuli. Those who experienced auditory perceptions sometimes performed callous acts indicating that they were responding to the instructions of the voices heard.^28^

Cultural and religious illnesses were also understood as a delusion where a person presented a fixed belief where a person display a strong belief of being followed for bad intention, people are jealous of him for having a lot of asserts, or of having special powers among other beliefs. Here, the spirit-possessed person displayed the belief of special powers such as healing or the ability of the mind to detect what the ancestors want. Some believe that they are prominent figures such as the president, Moses from the Bible or Jesus Christ/God.^29^ There was also DIDs that share many characteristics that one can associate with cultural and religious illnesses. A person with DID can display more than one personality in one person or live two lives such as a male who speaks with a female voice during trances or dresses like women. Cultural and religious illnesses cause a person to experience severe forgetfulness of who they are; some develop new identities, while others are in a state of ‘bewildered wandering’ called dissociative fugue.^30^

In Sepedi, dissociative fugue means *bokgolwa* and the person is *lekgolwa* [a missing person who has developed a new identity because of forgetfulness of who they are]. Al-Adawi et al.,^25^ Rashed^26^ and Goblirsch^31^ described DID as an alteration in identity called dissociative fugue in which a person lost awareness of their identity and/or other important autobiographical information. Forgetfulness led a person to be engaging in some form of unexpected travel.^32^

### Ancestral calling

Mental healthcare providers also considered cultural and religious illnesses an ancestral calling based on knowledge they acquired during their upbringing and socialisation. For the MHCPs who understood ancestral calling, they easily accommodated information presented by the spirit-possessed person and their family without judgement. The idea is that an ancestral spirit chooses a person from the family lineage and use them to take over their cultural healing practices with no choice left to those affected. Juro^33^ agreed that ancestral calling was a condition in which one or more spirits resided in the body of a human being and took control of the person. According to Ogana and Ojong,^34^ cultural and religious illnesses are an inborn gift in families. Letšosa,^35^ on the other hand, disagreed, instead maintaining that cultural and religious illnesses were bad spirits pretending to be God’s assistants, while Meveni^36^ reported that cultural and religious illnesses were not a gift but an anti-social and aggressive behaviour that normally caused social tension in the community.

### Cultural and religious illnesses as demonic power

Mental healthcare providers understood cultural and religious illnesses as a power that influenced a certain behaviour or action that a possessed person demonstrates. The demon is hosted in a person and manifest itself using the mouth of the possessed person to curse the target person. The cursed person could be aggressive and causes fights with the assistance of the demonic power. Some people are controlled by the demonic spirt to perform unacceptable acts that cause harm to humanity.^37^ A study conducted at Medunsa by Van Rensburg, Fourie and Pretorius^38^ indicated that some MHCPs understood that cultural and religious illnesses were a supernatural sorcery that could be the source of many illnesses. While Ndlovu^32^ revealed that the demons could speak through a possessed person. According to Juro,^39^ demonic power operated in such a way that a person’s normal way of living changed, and the demonic power could speak and act through them as their complete slave and instrument to perform evil deeds. On the contrary, Mothibe and Sibanda^40^ understood cultural and religious illnesses as a positive state where the ancestral spirit inhabited the person, serving as a channel for messages that needed to be delivered from ancestors to living family members.

### Cultural and religious illnesses as unclassified disorder

Mental healthcare providers understood cultural and religious illnesses as an unclassified disorder where some cultural and religious illnesses are not clearly described and defined. Not all manifestations observed and presented by the spirit-possessed person appear under DSM-5. Therefore, MHCPs could not classify cultural and religious illnesses in the list of diagnoses. Reeve, Sheaves and Freeman^41^ confirmed that other disorders did not always meet the full diagnostic criteria for classified diagnoses; hence, the use of provisional or final diagnosis as ‘not otherwise specified’, ‘other’ or ‘related’ diagnoses to cover up mental and related diagnosis such as cultural and religious illnesses. Kajawu, Chiweshe and Mapara^41^ revealed that some spirit-possessed persons present with some mental disorders that were not recognised from a biomedical point of view such as the supernatural, cultural or social problems. Therefore, the researchers suggested that cultural and religious illnesses should form part of healthcare services or workshops for MHCPs to understand and provide holistic care.

## Conclusion

The MHCPs’ understanding of cultural and religious illnesses is fourfold: mental illness, ancestral calling, demonic power and unclassified disorder. The MHCPs’ belief system, culture, religion or profession influence their understanding and interpretation of cultural and religious illnesses. It is recommended that, any illnesses that do not respond to psychiatric treatment be referred to traditional health practitioners and faith healers for further expert cultural and religious assessment and management.
